# Real-world assessment of attenuated dosing anti-PD1 therapy as an alternative dosing strategy in a high-income country (as defined by World Bank)

**DOI:** 10.3389/fonc.2022.932212

**Published:** 2022-11-17

**Authors:** Jia Li Low, Yiqing Huang, Kenneth Sooi, Zhi Yao Chan, Wei Peng Yong, Soo Chin Lee, Boon Cher Goh

**Affiliations:** ^1^ Department of Hematology-Oncology, National University Cancer Institute (NCIS), Singapore, Singapore; ^2^ Department of Pharmacy, National University Hospital, National University Health System, Singapore, Singapore; ^3^ Cancer Science Institute (CSI), National University Singapore, Singapore, Singapore; ^4^ Department of Medicine, Yong Loo Lin School of Medicine, National University of Singapore, Singapore, Singapore

**Keywords:** PDL1, attenuated, lung cancer, immunotherapy, immune check inhibitor (ICI), dose, non-small cell lung cancer

## Abstract

The rising cost of oncological drugs poses a global challenge to patients, insurers, and policy makers, with the leading drugs worldwide by revenue from immune checkpoint inhibitors (ICIs). Despite its cost, ICI is marked as a paradigm shift, offering the potential of a long-term cure. To reduce cost, an attenuated dose of ICI based on pharmacological principles can be used while maintaining efficacy. This real-world study aims to examine the prescribing patterns, the effect of financial constraints, and the outcomes in non-small cell lung cancer (NSCLC). All patients receiving palliative intent ICI treatment for advanced NSCLC between January 2014 and April 2021 in National University Hospital, Singapore were recruited. Demographics, prescription trends, factors affecting the prescription of attenuated dose ICI (AD ICI) *versus* standard dose ICI (SD ICI), and the effect of dose on survival outcomes, toxicities, and costs were examined. Two hundred seventy-four received ICI. The majority of them were treated in first-line setting. One hundred sixty-two (59%) of patients received AD ICI, whereas 112 (41%) received SD ICI. Patients who did not have a supplemental private as-charged health insurance plan were more likely to have received AD ICI (OR: 4.53 [2.69–7.61] *p* < 0.001). There was no difference in progression-free survival (PFS) and overall survival (OS)—adjusted HR 1.07 CI [0.76, 1.50] *p* = 0.697 and HR 0.95 CI [0.67, 1.34] *p* = 0.773, respectively, between patients who received AD *versus* SD ICI. A cost minimization analysis evaluating the degree of cost savings related to drug costs estimated a within study cost saving of USD 7,939,059 over 7 years. Our study provides evidence for AD-ICI as a promising strategy to maximize the number of patients who can be treated with ICI. This has the potential to make significant economic impact and allow more patients to benefit from novel therapies.

## Introduction

Breakthroughs in anti-cancer treatment have altered the treatment paradigm in oncology. However, the costs of treatment pose a global challenge to patients, insurers, and policy makers. Global sales of oncology drugs reached USD 176 billion in 2021. This is more than double that of the next most costly item, vaccines. By 2026, cancer drug sales are expected to almost double to USD 320.6 billion and approach 22% of the pharmaceutical market (1–4). The leading drugs worldwide by revenue currently comes from immune checkpoint inhibitors (ICIs) (5, 6).

Singapore is a high-income economy as defined by World Bank with a gross national income of USD 54,539 per capita. Singapore’s healthcare system is also ranked one of the best in Asia and the world, focusing on quality, efficiency, and cost (7). However, rising national health expenditures is receiving increasing attention. With cancer being the nation’s leading cause of death and rising cost of cancer drugs, the country’s spending on cancer drugs has grown at a compound annual growth rate of 20% between 2017 and 2021. This poses a challenge to the nation’s co-payment healthcare system. Singapore’s healthcare system revolves around a mixed financing system. The country’s public statutory insurance system, MediShield, is a basic insurance plan that covers a portion of hospitalization and outpatient treatment. This is complemented by government subsidies, as well as a compulsory savings account Medisave for each citizen, which pays for inpatient care and selected outpatient services (8–10).

Despite its cost, ICI targeting program death 1 (PD-1) and PD-ligand 1 (PD-L1) are marked as a paradigm shift in cancer treatment and offer the possibility of long-term survival (11–18). However, cost effectiveness and sustainability of these drugs are important issues to be considered in the real world (19, 20). Financial toxicity has not only shown to reduce quality of life, increase symptom burden, and potentially affecting survival of patients (21, 22), but it also threatens the financial sustainability of our healthcare system. The potential impact is the lack of access to drugs and benefits of novel therapies.

With the widespread use of ICI, these escalating healthcare costs are necessitating the practice of value-based oncology. An alternative strategy is the development of lower cost off-label treatment regimens, based on pharmacological rationale. This approach of interventional pharmacoeconomics seeks to decrease costs while maintaining equivalent efficacy (23, 24).

In our study, we looked at the real-world use of ICI in non-small cell lung cancers (NSCLC) in our institution since its approval in 2014 and examined the demographics, factors affecting the prescribing patterns, the effects of financial toxicity, and the survival outcomes of patients treated with ICI.

## Methods

### Patients and treatment

A retrospective cohort study was carried out for all patients receiving palliative intent ICI treatment for advanced NSCLC between January 2014 and April 2021 in an academic tertiary cancer center (National University Cancer Institute, Singapore; NCIS). NSCLC was selected as ICI has been widely approved for use. All patients were identified retrospectively. Patients receiving ICI and enrolled into clinical trials were excluded from the study. Baseline patient demographics, tumor, and treatment characteristics were extracted from electronic medical records. Local protocols continue treatment until disease progression, unacceptable toxicities, death, patient’s decision to stop treatment, or after 2 years of treatment, although some patients who remained progression free after 2 years continued treatment.

### Response evaluation

Chest and/or abdominal computed tomography (CT) scans were performed by clinicians every 8–12 weeks, as part of routine clinical care, to evaluate patient’s response and assess for disease progression. Progression-free survival (PFS) was measured from time of initiation of drug to disease progression by RECIST or death due to any cause. Overall survival (OS) was measured from time of initiation of drug to death due to any cause. Safety analysis examined the incidence of ≥ Grade 3 immune-related adverse events (irAEs) and adverse events (AEs) as recorded by clinicians.

### Statistics and economic analysis

Continuous and categorical variables were summarized as median (inter-quartile range) and frequency (percentage), respectively. The differences in baseline characteristics of patients receiving attenuated dose ICI (AD ICI) and standard dose ICI (SD ICI) were evaluated using the multinomial logistic regression model. SD ICI was defined as the FDA-approved dose of pembrolizumab 200 mg every 3 weeks or 400 mg every 6 weeks, nivolumab 240 mg every 2 weeks, or 480 mg 4 weeks, atezolizumab 1200 mg every 3 weeks, and durvalumab 10 mg/kg every 2 weeks. AD ICI was defined as a lower than FDA-approved dose of ICI. In our study, AD ICI was given based on an approximate 2 mg/kg weight-based dose of pembrolizumab and 3 mg/kg weight-based dose of nivolumab. The differences in toxicities of the two doses of ICI were tested using the chi-square or Fisher’s exact test whenever applicable.

We also plotted the Kaplan–Meier curve to find a difference in PFS and OS between the AD ICI and SD ICI. Univariate and multivariable Cox proportional hazard regression model was used to find variables associated with PFS and OS in this population. Quantitative association from Cox regression was expressed as hazard ratio (HR) with its corresponding 95% confidence interval (CI). All the tests used in this study were two sided, and *P*-values < 0.05 were considered as statistically significant. All these tests were performed using Stata version 17.

Based on an acceptance of non-inferior survival and toxicity outcomes, a limited economic evaluation was carried out using a cost-minimization approach (25). This assessed the monetary savings available from the use of AD ICI instead of SD ICI across the entire study population based on the total cycles received by the study population and price of ICI. A fixed price of ICI was assumed. Sensitivity analysis considered the potential savings within the study population if all patients were to receive AD ICI. The dose of AD ICI for this analysis was calculated at pembrolizumab 100 mg and nivolumab 180 mg based on an approximate weight-based dosing of 2 and 3 mg/kg, respectively, vial size and median weight of 56 kg in our population. Given the identical regimens and observed clinical outcomes, all other costs were assumed to remain constant. This analysis was only performed for patients receiving pembrolizumab and nivolumab, as none of the patients who received durvalumab and atezolizumab were treated at attenuated dose.

## Results

### Patient characteristics

Two hundred seventy-four patients received immunotherapy in for advanced NSCLC from 2014 to April 2021 at NCIS. Baseline demographics are shown in **Table 1**. Median age was 65.1 (range: 28.3–92.2). Majority of the patients were Chinese (214, 78%), Singaporeans (239, 87%), men (202, 73%), had an ECOG status of 0/1 (236, 86%), were current/ex-smokers (177, 65%), married (240, 88%), had children (240, 88%), and worked in the service and sales sector (879, 29%) according to the International Standard Classification of Occupations (ISCO) 8 structure. The average body weight was 56 kg (range: 31–103).

**Table 1:** Patient demographics.

		Total	Attenuated dose ICI	Standard dose ICI	Odds ratio (95% CI)	*P*-value
		274 (100%)	162 (59%)	112 (41%)		
**Median age (range)**		65 (28-92)	67 (43-92)	62 (28-80)	1.05 [1.02, 1.07]	*p* = 0.001
**Median weight (range)**		56 (31-103)	54 (31-83)	65 (38-103)	0.94 [0.91, 0.96]	*p* < 0.001
**Ethnicity**	*Chinese*	214 (78%)	132 (81%)	82 (73%)	1.18 [0.79, 1.76]	*p* = 0.420
	*Malay*	32 (12%)	20 (12%)	12 (11%)		
	*Indian*	9 (3%)	6 (4%)	3 (3%)		
	*Others*	19 (7%)	4 (2%)	15 (13%)		
**Nationality**	*Singaporean*	239 (87%)	150 (93%)	89 (79%)	1.18 [0.44, 3.19]	*p* = 0.743
	*Singaporean PR*	10 (4%)	5 (3%)	5 (4%)		
	*Foreigner*	25 (9%)	7 (4%)	18(16%)		
**Gender**	*Female*	72 (26%)	46 (28%)	26 (23%)	0.76 [0.44, 1.33]	*p* = 0.339
	*Male*	202 (73%)	116 (72%)	86 (77%)		
**ECOG**	*0/1*	236 (86%)	134 (83%)	102 (91%)	1.30 [0.81, 2.08]	*p* = 0.280
	*2*	20 (7%)	17 (11%)	3 (3%)		
	*3*	17 (6%)	10 (6%)	7 (6%)		
	*Unknown*	1 (1%)	1 (1%)	0 (0%)		
**Smoking status**	*Current smoker*	97 (36%)	58 (36%)	39 (35%)	0.97 [0.73, 1.28]	*p* = 0.819
	Ex-smoker	80 (29%)	49 (30%)	31 (28%)		
	Never smoker	92 (33%)	51 (32%)	41 (37%)		
	Unknown	5 (2%)	4 (2%)	1 (1%)		
**Marriage status**	*Married*	240 (88%)	140 (86%)	100 (89%)	1.064 [0.72, 1.58]	*p* = 0.760
	*Divorced/Separated*	10 (4%)	7 (4%)	3 (3%)		
	*Single*	22 (8%)	15 (9%)	7 (6%)		
	*Unknown*	2 (1%)	0 (0%)	2 (2%)		
**Have children**	*Yes*	240 (88%)	147 (91%)	93 (83%)	0.45 [0.25, 0.81]	*p* = 0.008
	*No*	26 (9%)	15 (9%)	11 (10%)		
	*Unknown*	8 (3%)	0 (0%)	8 (7%)		
**Paying class**	Private	60 (22%)	18 (11%)	42 (38%)	0.21 [0.11, 0.39]	*p* < 0.001
	*Subsidized*	214 (78%)	144 (89%)	70 (63%)		
**Medisave**	Yes	234 (85%)	144 (89%)	70 (62%)	0.31 [0.16, 0.63]	*p* = 0.001
	No	40 (15%)	18 (11%)	42 (38%)		
**MediShield**	Yes	244 (89%)	153 (94%)	91 (81%)	0.26 [0.11, 0.58]	*p* = 0.001
	No	30 (11%)	9 (6%)	21 (19%)		
**Supplemental as charged private health insurance**	Yes	105 (38%)	39 (24%)	66 (59%)	4.53 (2.69-7.61)	*p* < 0.001
	No	169 (62%)	123 (76%)	46 (41%)		
**Occupation (ISCO-8 structure)**	Manager	28 (10%)	10 (6%)	18 (16%)	1.02 [0.95, 1.09]	*p* = 0.602
	Professionals	44 (16%)	21 (13%)	23 (21%)		
	Technician	6 (2%)	4 (2%)	2 (2%)		
	Clerical support workers	6 (2%)	5 (3%)	1 (1%)		
	Service and sales	79 (29%)	55 (34%)	24 (21%)		
	Skill, agricultural, forestry, and fishery workers	2 (1%)	1 (1%)	1 (1%)		
	Craft and related trades workers	5 (2%)	3 (2%)	2 (2%)		
	Plant and machine operator	19 (7%)	10 (6%)	9 (8%)		
	Elementary operations	23 (8%)	14 (9%)	9 (8%)		
	Arm forces operation	2 (1%)	0 (0%)	2 (2%)		
	Unemployed	18 (7%)	14 (9%)	4 (4%)		
	Unknown	42 (15%)	26 (15%)	17 (15%)		

In terms of healthcare services, most patients were government subsidized (214, 78%), had Medisave (234, 85%), had MediShield (244, 89%), and did not have a supplemental private health insurance plan (169, 62%).

Treatment characteristics are summarized in **Table 2**. The majority of the patients received pembrolizumab (229, 84%), received ICI monotherapy (164, 60%), and were treated in the first-line setting (169, 62%).

**Table 2:** Treatment characteristics.

		Lung cancer (*N* = 274)		
		Total	Attenuated dose ICI (*n* = 162)	Standard dose (*n* = 112)
Histology	Squamous	43 (16%)	26 (16%)	17 (15%)
	Non-squamous	231 (84%)	136 (84%)	95 (84%)
Tumor PDL1 TPS	0%	*51 (19%)*	29 (18%)	23 (21%)
	1-49%	*57 (21%)*	28 (17%)	29 (26%)
	≥50%	*103 (38%)*	68 (42%)	35 (31%)
	Unknown	62 (23%)	37 (23%)	25 (22%)
EGFR	Positive	*27 (9%)*	14 (9%)	13 (12%)
	Negative	247 (84%)	148 (91%)	99 (88%)
ALK	Positive	3 (1%)	2 (1%)	1 (1%)
	Negative	271 (92%)	160 (99%)	111 (99%)
ROS	Positive	3 (1%)	0 (0%)	3 (3%)
	Negative	271 (92%)	162 (100%)	109 (97%)
Line of treatment in the palliative setting	First line	169 (62%)	98 (60%)	71 (63%)
	Second line	72 (26%)	44 (27%)	28 (25%)
	Third line	20 (7%)	11 (7%)	9 (8%)
	Fourth line and beyond	13 (5%)	9 (6%)	4 (4%)
Partner drug	Monotherapy	164 (60%)	115 (71%)	51 (45%)
	Combined with chemotherapy	110 (40%)	47 (29%)	61 (54%)
Type of immunotherapy used	Pembrolizumab	229 (84%)	142 (88%)	87 (78%)
	Nivolumab	31 (11%)	20 (12%)	11 (10%)
	Atezolizumab	12 (4%)	0	12 (11%)
	Durvalumab	3 (1%)	0	2 (2%)
Median dose of immunotherapy (mg/kg)	Pembrolizumab	2.22 (1.20-4.98)	1.92 (1.20-3.23)	2.99 (1.94-4.98)
	Nivolumab	3.01 (2-8.18)	2.86 (2-3.18)	4 (2.63-8.18)
	Atezolizumab	17.91 (13.17-27.27)	–	17.91 (13.17-27.27)
	Durvalumab	10	–	10

**Figure 1:**
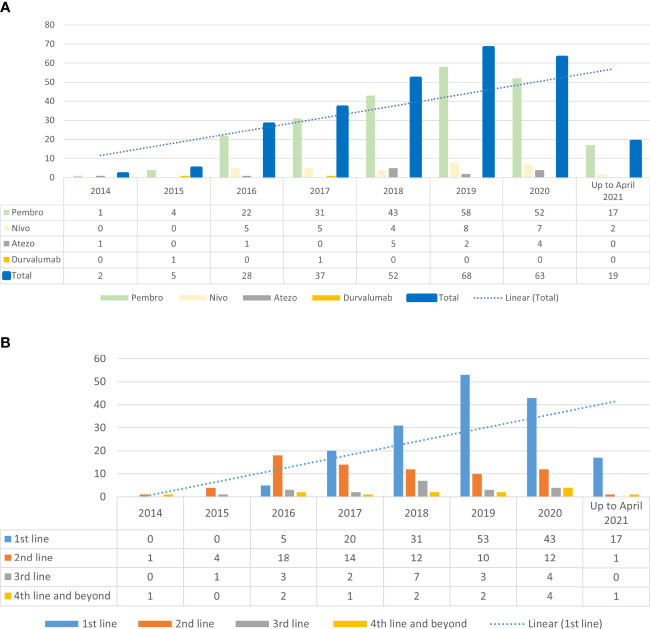
**(A)** Trend of immune checkpoint inhibitor use since 2014 **(B)** Immune checkpoint inhibitor and line of treatment.

**Figures 1A, B** illustrate the increasing trend of ICI usage in our study population since its approval in 2014 and the shift in the use of ICI in first-line setting, respectively. One hundred sixty-two (59%) of patients received AD-ICI, whereas 112 (41%) received SD-ICI. Using the multinomial logistics regression model, we found that patients who did not have a supplemental private as-charged health insurance plan were more likely to have received LD-ICI (OR: 4.53, 95% CI [2.69, 7.61] *p* < 0.001) (**Table 1**).

### Survival analysis

All patients were included in the survival analysis. Median follow-up duration was 25.1 months.

All variables were analyzed to find independent variables associated with PFS (**Table 3**) and OS (**Table 4**). Univariate analysis showed that male gender and a heavier weight were associated with improved PFS, whereas a poorer ECOG status and a later line of treatment were associated with a decreased PFS. For OS, foreigners, heavier weight, and private-paying patients was associated with an improved OS, whereas a poorer ECOG status and a later line of treatment were associated with a decreased OS.

**Table 3:** Univariate and multivariate Cox regression for progression free survival.

		Univariate analysis			Multivariate analysis		
Characteristics		HR	95% CI	*p*-value	HR	95% CI	*p*-value
Age		1.00	[0.99, 1.013]	0.916			
Ethnicity (reference: Chinese)	Malays	1.305	[0.86, 1.97]	0.210			
	Indians	0.84	[0.34, 2.05]	0.699			
Race (reference: Singaporean)	Singapore PR	1.32	[0.62, 2.8]	0.476			
	Foreigners	.68	[0.39, 1.20]	0.184			
Male gender		0.72	[0.53, 0.96]	0.028	0.973	[0.68, 1.37]	0.846
ECOG (reference: ECOG 0/1)	2/3	2.16	[1.69. 2.75]	*p* < 0.001	2.29	[1.77, 2.96]	*p* < 0.001
Smoking status (reference: current smokers)	Former smoker	0.82	[0.58, 1.16]	0.266	1.12	[0.93, 1.37]	0.246
	Non-smokers	1.33	[0.96, 1.85]	0.084			
Weight		0.99	[0.98, 1.00]	0.045	1.00	[0.98, 1.01]	0.309
Private patient		0.79	[0.56, 1.13]	0.204			
No private as charge insurance		0.93	[0.71, 1.23]	0.623			
PDL1 TPS score (reference: PDL1 0%)	PDL1 1-49%	1.23	[0.80, 1.899]	0.352			
	PDL1 >/= 50%	1.00	[0.67, 1.49]	0.995			
Line of treatment	2nd line	1.53	[1.11, 2.10]	0.009	1.50	[1.25, 1.79]	*p* < 0.001
	3rd line	1.77	[1.09, 2.88]	0.021			
	4th line and beyond	3.06	[1.55, 6.07]	*p* < 0.001			
Immunotherapy combined with chemotherapy (reference: immunotherapy alone)		0.70	[0.53, 0.94]	0.015	0.98	[0.70, 1.36]	0.882
Driver mutation positive (reference: driver mutation negative)		1.92	[1.29, 2.87]	0.001	1.14	[0.703, 1.85]	0.593
Attenuated dose immunotherapy (reference: standard dose immunotherapy)		1.21	[0.91, 1.61]	0.183	1.07	[0.76, 1.50]	0.697

**Table 4:** Univariate and multivariate Cox regression for overall survival.

		Univariate analysis			Multivariate analysis		
Characteristics		HR	95% CI	*p*-value	HR	95% CI	*p*-value
Age		1.018	[1.00, 1.03]	0.056			
Ethnicity (reference: Chinese)	Malays	1.06	[0.68, 1.64]	0.804			
	Indians	.421	[0.13, 1.31]	0.133			
Nationality status (reference: Singaporean)	Singapore PR	1.32	[0.54, 3.23]	0.537	0.57	[0.35, 0.92]	0.021
	Foreigners	0.19	[0.059, 0.58]	0.004			
Male gender		0.81	[0.58, 1.129]	0.199			
ECOG (reference: ECOG 0/1)	2/3	2.26	[1.77, 2.88]	*p* < 0.001	2.22	[1.72, 2.87]	< 0.001
Smoking status (reference: current smokers)	Former smoker	0.83	[0.57, 1.20]	0.315			
	Non-smokers	1.00	[0.69, 1.42]	0.959			
Weight		0.98	[0.97, 0.99]	0.004	0.99	[0.98, 1.00]	0.055
Private patient		0.58	[0.38, 0.89]	0.013	0.90	[0.56, 1.45]	0.654
No private as charge insurance		1.06	[0.79, 1.44]	0.686			
PDL1 TPS score (reference: PDL1 0%)	PDL1 1-49%	1.26	[0.78, 2.03]	0.354			
	PDL1 >/= 50%	1.12	[0.72, 1.74]	0.612			
Line of treatment	2nd line	1.26	[0.89, 1.78]	0.185	1.45	[1.23, 1.71]	p<0.001
	3rd line	1.29	[0.75, 2.23]	0.356			
	4th line and beyond	2.27	[1.15, 4.51]	0.019			
Immunotherapy combined with chemotherapy (reference: immunotherapy alone)		0.74	[0.54, 1.01]	0.060			
Driver mutation positive (reference: Driver mutation negative)		1.31	[0.84, 2.05]	0.236			
Lower dose immunotherapy (reference: approved dose immunotherapy)		1.34	[0.99, 1.83]	0.060	0.95	[0.67, 1.34]	0.773

Multivariate logistic regression analysis was conducted to elucidate associations between significant variables found in univariate analysis between PFS and OS. Only a poorer ECOG status and a later line of treatment continued to be associated with both a decreased PFS and OS.

**Figure 2:**
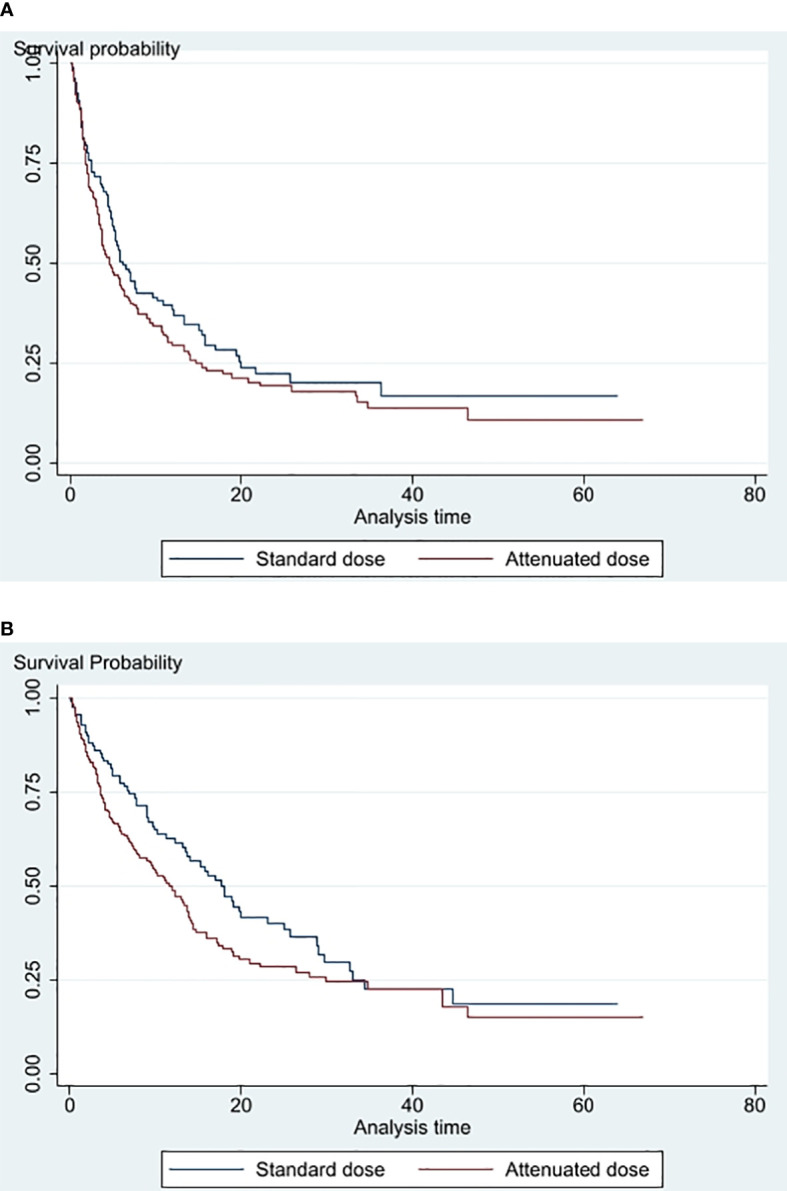
**(A)** PFS of standard dose vs attenuated dose immune checkpoint inhibitors **(B)** Overall survival of standard dose versus attenuated dose immune checkpoint inhibitors.

The Kaplan–Meier curves for PFS and OS are demonstrated in **Figures 2A, B**. The median PFS and OS for AD ICI and SD ICI were 4.6 and 6.1 months and 11.9 and 17.9 months, respectively. The univariate Cox regression model demonstrates no significant difference in PFS (raw HR 1.21, 95% CI [0.91, 1.61], *p* = 0.183, and OS (raw HR 1.34, 95% CI [0.99, 1.83], *p* = 0. 0.060). When adjusted for significant variables found in the univariate analysis, the multivariate Cox regression model shows no significant difference in PFS (adjusted HR 1.07, 95% CI [0.76, 1.50], *p* = 0.843) and OS (adjusted HR 0.95, 95% CI [0.67, 1.34], *p* = 0.773) between AD ICI and SD ICI.

### Toxicities

Thirty-seven (12%) of the patients discontinued treatment because of toxicities. There was no dose relationship between ICI and serious irAEs or deaths. The rates of G3 or more AEs and deaths were 10% *versus* 18% (*p* = 0.056) and 3% and 4% (*p* = 0. 0.386) for AD and SD treatments, respectively (**Table 5**).

**Table 5:** Toxicity analysis of attenuated dose *versus* standard dose immune checkpoint inhibitors.

	Total	Attenuated dose ICI (*n* = 165)	Standard dose (*n* = 109)	*P*-value
No. of patients with G3 or more adverse events	37 (14%)	17 (10%)	20 (18%)	0.056
No. of patients who discontinued treatment because of irAE	32 (12%)	15 (9%)	17 (16%)	0.101
No. of deaths from irAE	8 (3%)	6 (4%)	2 (2%)	0.386

### Treatment costs

In our study, a lower than FDA-approved dose of ICI was routinely delivered based on an approximate 2 mg/kg weight-based dose of pembrolizumab and 3 mg/kg weight-based dose of nivolumab for patients who did not have adequate financial reimbursement plan or based on physician’s preference. None of the patients who received durvalumab or atezolizumab received a lower than approved dose (**Table 2**).

In our institution, a 100-mg vial of pembrolizumab costs USD 3,778, whereas a 100- and 40-mg vial of nivolumab costs USD 976 and 433, respectively, in Singapore. The total number of cycles of pembrolizumab and nivolumab received in our study was 3,743, median cycles 8 and 7, respectively. Cycles (2,313 *vs.* 1,430) were delivered in the AD ICI and SD ICI groups. We estimated a total cost savings in our study population of USD 7,939,059 based on the total number of cycles of ICI received in the AD ICI group. This translates to cost savings per cycle for each patient of USD 3,778 and USD 433 for pembrolizumab and nivolumab, respectively.

The cost minimization analysis demonstrates a cost saving of USD 12,863,264 if a weight-based dose of AD ICI was used instead of SD ICI. This would translate to a cost saving of USD 55,692 and USD 5,335 per patient receiving pembrolizumab and nivolumab, respectively (**Table 6**).

**Table 6:** Cost analysis of attenuated dose *versus* standard dose pembrolizumab and nivolumab.

		Pembrolizumab		Nivolumab	
	Total	Attenuated dose ICI (*n* = 142)	Standard dose ICI (*n* = 86)	Attenuated dose ICI (*n* = 20)	Standard dose ICI (*n* = 11)
Total number of cycles received	3743	2074	1287	239	143
Median number of cycles		8		7	
Cost (USD)	18,237,950	7,835,572	9,724,572	336,751	341,055
In study savings (USD)	7,939,059	7,835,572	–	103,487	–
In study savings (USD)/cycle	–	3778	–	433	
Amount of savings if attenuated used for all patients (USD)	12,863,264	7,835,572	4,862,286	103,487	61,919

## Discussion

To our knowledge, our study represents the largest cohort to date to evaluate the real-world use of ICI and the efficacy of an attenuated dose of ICI in NSCLC.

The overall use of ICI and the use in the first-line setting have increased over the years in our institution since its approval in 2014 for use in NSCLC, which is reflective of the global trend (26–28). The majority of patients also received ICI upfront in their treatment, in line with FDA’s approval of ICI in NSCLC (29).

However, 162 (59%) of patients in our institute did not receive SD ICI. Only 105 (38%) of the patients had a supplemental as-charged private insurance plan on top of Singapore’s public statutory insurance system, and this was significantly associated with the use of SD ICI with odds ratio of 4.53. Despite financial barriers to prescribing SD ICI, multivariate analysis showed no significant differences in PFS and OS despite the discrepancy in the doses of ICI with an adjusted HR of 1.07 and 0.95, respectively. Only a poorer ECOG status and treatment in later lines were significantly associated with both a poorer PFS and OS, which were within expectations.

Pharmacological principles for dose reduction and weight-based dosing were employed for patients who did not have adequate financial reimbursement. It is known that there are nonlinear relationships between dose of ICI and clinical outcomes. The pharmacokinetic analysis of doses of 200 mg and 2 mg/kg of pembrolizumab has shown similar exposure distributions with no advantage to either dosing approach. Pembrolizumab kinetics has also shown that there is 95% trough target engagement with dosing of 0.8 mg/kg every 3 weeks with saturation of PD-1 receptors at a dose of ≥1 mg/kg. Similarly, for nivolumab, a dose ranging phase 1b study showed that PD-1 receptor occupancy was already saturated at a dose of 0.3 mg/kg (30–38). In our study, the median dose of patients receiving AD ICI was close to 2 and 3 mg/kg for pembrolizumab and nivolumab, respectively. This could explain why we did not see an efficacy difference between the AD ICI and SD ICI.

A weight-based dosing of ICI also appears to be cost efficient. Goldstein et al. demonstrated huge cost savings to the U.S. healthcare system by using a personalized dosing of 2 mg/kg of pembrolizumab (20). In our study population, an estimated in study cost savings was USD 8,154,100. This could increase to USD 13,207,243 if all patients received AD ICI. Other than cost savings, adoption of a weight-based dosing approach will also decrease the dosage drugs needed and may allow more global access to effective yet value-driven therapeutics. While the development of ICI has improved the survival of people with several kinds of cancer, it is not available to most people in low- and middle-income countries (39). In fact, while the importance of immune-oncology drugs was recognized, it is not listed in the World Health Organisation essential medical list (WHO EML) at the 23^rd^ WHO meeting on essential medicines held in September 2021 due to their high cost (40). In a study to evaluate the concordance of medications included in the WHO EML and availability on the frontline of clinical care, striking barriers to accessing high-priority medicines in low- and middle- income countries remain. Core medications such as doxorubicin, cisplatin, and tamoxifen continue to be associated with risks of catastrophic out-of-pocket expenditure (41). The fact that substantial proportion cannot even afford older generic cytotoxic drugs, let alone ICI, highlights a major barrier in access to core medicines. The result of our study reinforces the sustainability and efficacy of use of weight-based dosing approach and may be a step toward addressing the affordability of oncology drugs, allowing more uniform global access to effective yet value-driven therapeutics.

Our study has its limitations. The PFS and OS were numerically better in SD ICI group but the retrospective nature of the study, differing baseline characteristics and limited sample size does not allow for valid efficacy comparison among different dosing strategies. In addition, the relatively small sample size limits the power of the study to demonstrate a statistically significant difference. Given the uncertainty of clinical outcome between the 2-dose groups, a prospective randomized controlled clinical trial is needed to clarify this. The use of SD-ICI was more likely in patients who had a supplemental as-charged private insurance plan on top of Singapore’s public statutory insurance system. This is a potential source of bias due to a positive relationship between health insurance coverage and health-related outcomes (42, 43). Other ICI such as tislelizumab, a China-developed anti-PD1 antibody, has also shown improve PFS in advanced non-squamous NSCLC when combined with chemotherapy (44) and was also reported to be cost effective (45) but is not yet approved or available in Singapore and, hence, not used in this study. Data to support the use of these newer anti-PD1 antibodies to the currently approved ones will also take time to accumulate. Finally, given no differences were identified in the clinical outcomes of the two regimens, a cost minimization analysis was used to examine the cost savings provided by AD ICI. This was not pre-planned and simply provides an indication of cost savings. The costs assessed are only those of the drug and do not include regimen-related costs such as drug administration, pre-medications, clinic visits, subsequent therapy, and management of AEs. While the costs are not anticipated to vary based on the study outcomes, further formal assessment of cost utility of AD ICI should be considered alongside future prospective randomized study.

Despite these limitations, our study reflects the real-world application of ICI where cost is prohibitive, outside the controlled setting of conventional clinical trials (39). It also suggests the efficacy of an attenuated dose of ICI, which can provide considerable cost savings to both patients and the healthcare system.

## Conclusion

Increasing cost of drugs contributes to the increasing cost of healthcare. This problem needs to be urgently tackled. Our real-world study demonstrates efficacy of AD ICI, based on a pharmacological rationale, which has the potential to make significant economic impact yet allow our patients to benefit from novel therapies. With the expanding role of ICI in various tumor types, this value driven approach will be highly relevant to patients, oncologists, and policy makers.

## Data availability statement

The original contributions presented in the study are included in the article/supplementary material. Further inquiries can be directed to the corresponding author.

## Ethics statement

The studies involving human participants were reviewed and approved by National Health Group Domain Specific Review Board (NHG DSRB) (Reference number: 2017/012654). Written informed consent for participation was not required for this study in accordance with the national legislation and the institutional requirements.

## Author contributions

JL, YH, KS, and ZC participated in data acquisition and collection. JL analyzed the data. JL, WY, SL, and BG contributed to manuscript writing. All authors contributed to the article and approved the submitted version.

## Funding

This research is supported by the Singapore Ministry of Health’s National Medical Research Council under its NMRC Centre Grant Programme CGAug16M005.

## Conflict of interest

The authors declare that the research was conducted in the absence of any commercial or financial relationships that could be construed as a potential conflict of interest.

## Publisher’s note

All claims expressed in this article are solely those of the authors and do not necessarily represent those of their affiliated organizations, or those of the publisher, the editors and the reviewers. Any product that may be evaluated in this article, or claim that may be made by its manufacturer, is not guaranteed or endorsed by the publisher.
